# Minimally Invasive Approach to the Management of Recurrent Spontaneous Hemarthrosis After Total Knee Replacement: A Report of Two Cases

**DOI:** 10.7759/cureus.69485

**Published:** 2024-09-15

**Authors:** Aysha Rajeev, Saurav Krishnan, George Koshy, Nathan Rajeev, Kiran Singisetti

**Affiliations:** 1 Trauma and Orthopaedics, Gateshead Health Foundation NHS Trust, Gateshead, GBR; 2 General Medicine, Gateshead Health Foundation NHS Trust, Gateshead, GBR; 3 Emergency Medicine, Gateshead Health Foundation NHS Trust, Gateshead, GBR

**Keywords:** arterial, embolization, recurrent, spontaneous, total knee replacement

## Abstract

Spontaneous recurrent hemarthrosis of the knee after total knee arthroplasty is an infrequent complication. Early recognition and prompt diagnosis are essential to avoid issues such as joint stiffness, chronic pain, and limited mobility. Conservative treatment methods are often effective. However, in cases where the bleeding recurs, imaging studies like CT or MRI angiograms are necessary to confirm the diagnosis and guide treatment.

We discuss two instances of spontaneous recurrent hemarthrosis that appeared six months and two years after total knee arthroplasty, both characterized by painful knee swelling and movement restriction. Following conservative management, both patients underwent CT angiograms and selective embolization, leading to excellent recovery without recurrence.

In summary, selective arterial embolization of the genicular arteries is a minimally invasive, safe, and effective procedure for treating recurrent spontaneous hemarthrosis after total knee replacement.

## Introduction

The incidence of recurrent hemarthrosis after total knee replacement ranges from 0.1% to 1.6% [1]. The sequelae of recurrent hemarthrosis in knee replacement can include pain, joint stiffness, reduced range of motion, and an increased risk of infection [2]. The time of occurrence can vary between two months to 18 years post-surgery [3]. The etiology can be attributed to either general or local causes. General causes include coagulopathies, anticoagulation agents, and hemophilia. Local causes may involve direct vascular injury, arteriovenous fistula, pseudoaneurysm, pigmented villonodular synovitis, synovial proliferation, impingement, or mechanical factors [4-7]. Prompt diagnosis and a targeted treatment approach are essential in managing hemarthrosis to prevent long-term complications.

We report two cases of recurrent spontaneous hemarthrosis after total knee replacement, with presentations occurring between six months and two years post-surgery. Both cases were investigated using CT angiography and successfully treated with a minimally invasive approach involving targeted selective embolization, resulting in excellent outcomes.

## Case presentation

Case report 1

**Figure 1:**
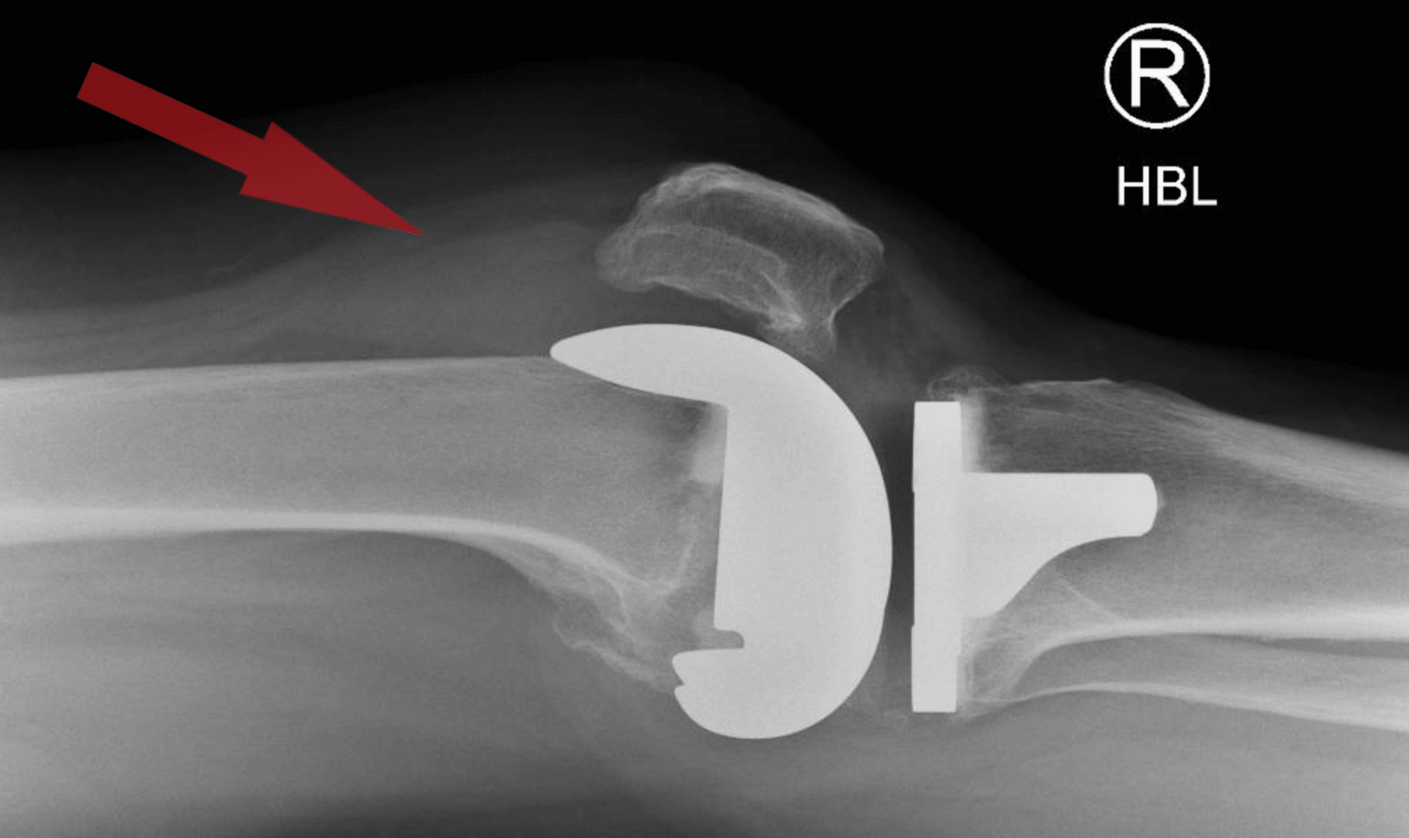
Lateral view of the knee of case 1 showing suprapatellar hemarthrosis in total knee replacement.

A 72-year-old woman underwent a right total knee replacement for severe osteoarthritis in 2018. She had no significant medical history. Postoperatively, she made an excellent recovery, achieving knee movement from 0 to 120 degrees of flexion with a stable knee. Two years after the surgery, she noticed swelling and pain in her right knee, along with restricted movement. On examination, there was effusion in the knee, generalized tenderness, and the range of motion was reduced to 10 to 90 degrees of flexion. X-ray examination of the knee showed evidence of hemarthrosis with the prosthesis in a satisfactory position (Figure 1). Blood tests revealed normal hemoglobin, platelet count, and liver function tests. The coagulation profile showed prothrombin time (PT) at 13 seconds (normal: 11-15 seconds), activated partial thromboplastin time (APTT) at 27.9 seconds (normal: 25-35 seconds), and Clauss fibrinogen at 4.49 g/L (normal: 1.5-5.0 g/L). Knee aspiration yielded frank blood, and culture and sensitivity tests were negative.

Initially, the patient was treated with knee splinting, ice application, and elevation, but the symptoms persisted. After discussing options with the patient, a decision was made to proceed with an angiogram and selective embolization. The procedure was performed by an experienced vascular interventional radiologist. After infiltrating 8 mL of 1% lidocaine in the right groin, the right common femoral artery (CFA) was punctured under ultrasound guidance, and a 5F sheath was introduced.

**Figure 2:**
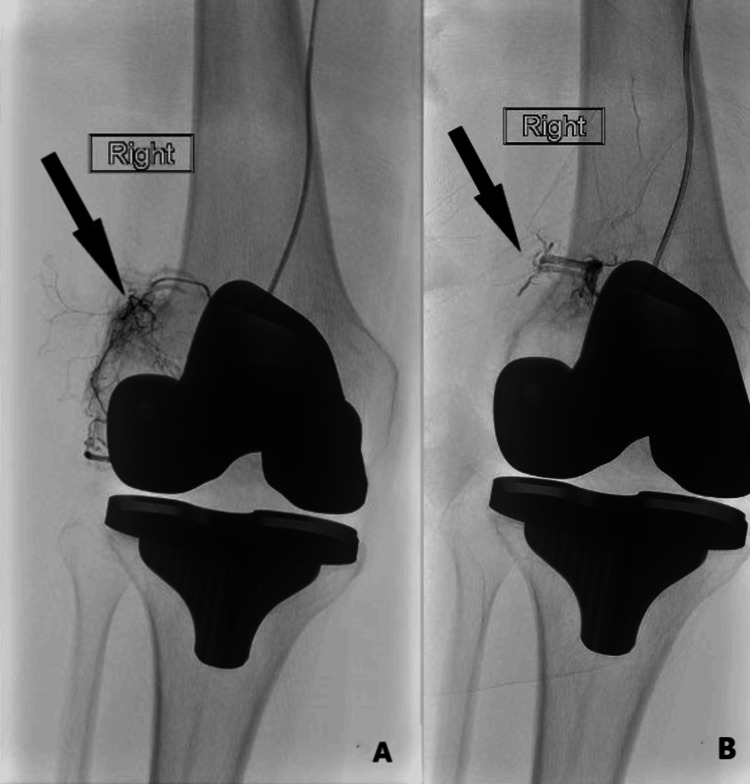
Pre- and post-embolization images of case 1. A Pre-embolization image showing “synovial blush” appearance in the lateral genicular arteries. B Post-embolization image showing blood flow stasis in the target area.

CT angiography revealed a synovial blush in the region of the lateral genicular artery. Selective embolization of a superolateral branch was performed using 350- to 500-micron polyvinyl alcohol (PVA) particles. The angiogram showed significant improvement (Figures 2A, 2B). The patient had an uneventful recovery and was discharged the same day. One year following embolization, she has had no further recurrences of hemarthrosis, regained full function, and achieved a range of motion of 0-115 degrees.

Case report 2

**Figure 3:**
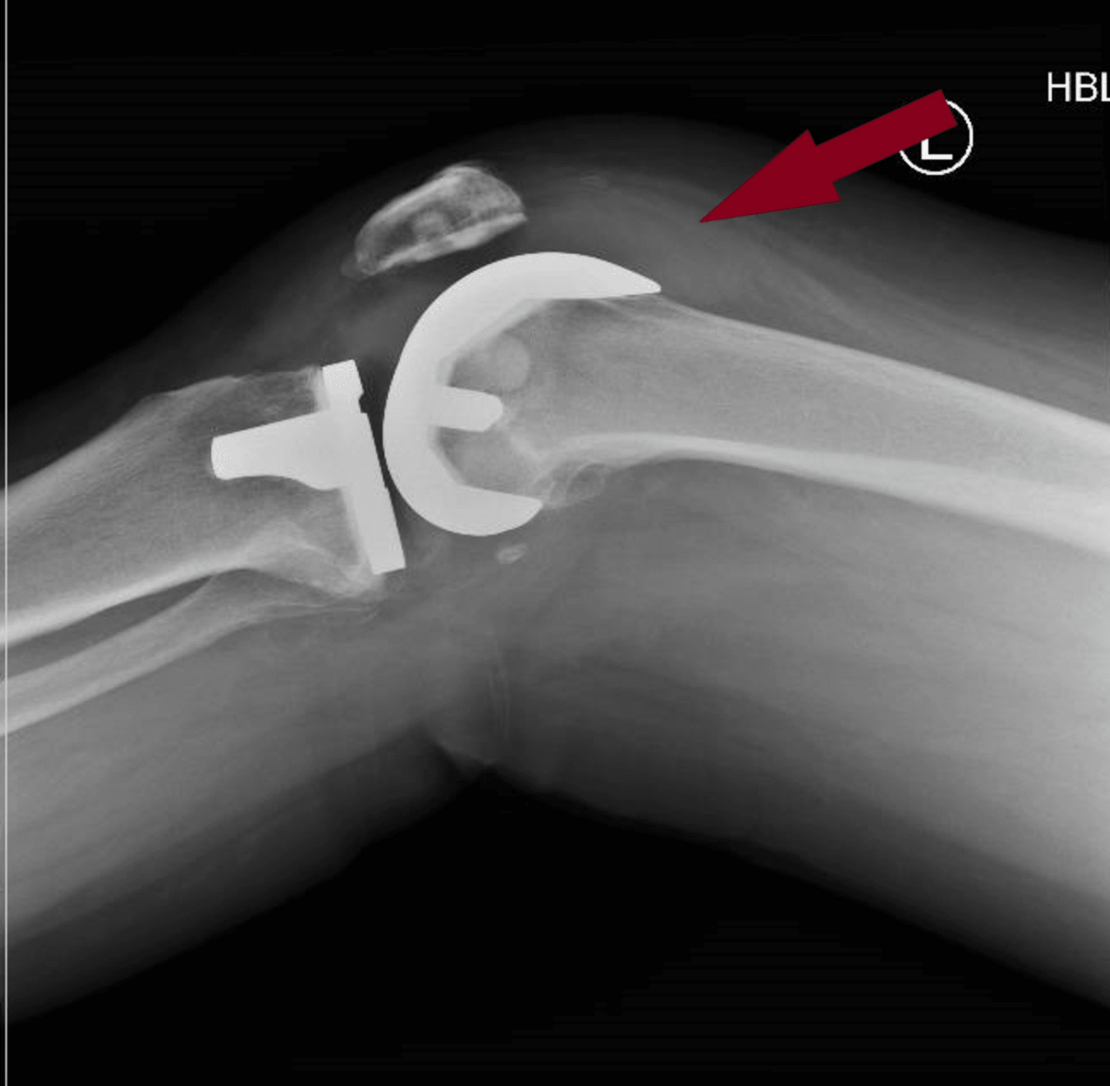
Lateral view of the left knee of case 2 showing suprapatellar hemarthrosis.

A 70-year-old woman underwent a left total knee replacement for osteoarthritis in 2021. Her past medical history included a pacemaker implantation for an irregular and slow heart rate, and she was on rivaroxaban (an oral anticoagulant). Postoperatively, she experienced knee stiffness, which required manipulation under general anesthesia, followed by continuous passive motion and intensive physiotherapy. She made a good recovery, with knee movement ranging from 0 to 115 degrees of flexion. However, six months after the operation, she noticed painful swelling of the left knee with limited movement. On examination, there was clinical evidence of effusion, and the range of motion was reduced to 20 to 80 degrees of flexion. X-ray examination showed evidence of hemarthrosis with the prosthesis in a satisfactory position (Figure 3). Blood tests, including full blood count, hemoglobin, platelet count, and liver function tests, were normal. However, the coagulation profile was deranged, with PT at 35.1 seconds (normal: 11-15 seconds), APTT at 48.5 seconds (normal: 25-35 seconds), and Clauss fibrinogen at 3.39 g/L (normal: 1.5-5.0 g/L).

**Figure 4:**
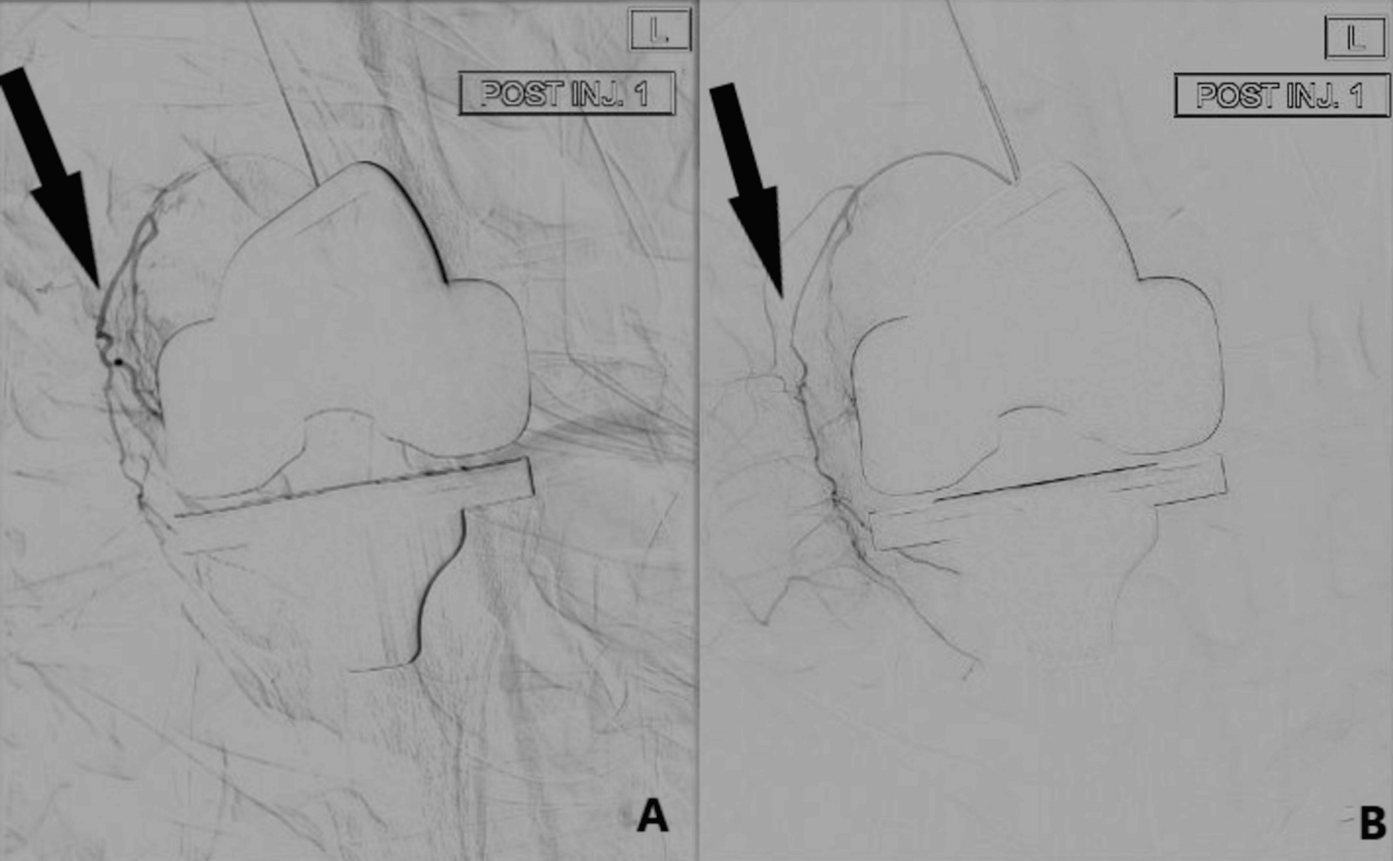
Pre- and post-embolization pictures of case 2. A Pre-embolization image showing “tumour blush” appearance in the medial genicular arteries. B Post-embolization image showing disappearance of the “tumour blush”.

The patient underwent a trial of conservative treatment, which included discontinuing rivaroxaban for a few weeks, along with rest, ice application, and elevation, but the symptoms did not resolve. After thorough discussion with the patient, the decision was made to perform an angiogram with selective embolization. Following the infiltration of 10 mL of 1% lidocaine in the left groin, the left CFA was punctured under ultrasound guidance, and a 5F sheath was introduced. Detailed angiography of the knee prosthesis revealed hypervascularity in the branches of the medial superior geniculate artery. This arterial branch was selectively catheterized and embolized to pre-stasis using 500- to 700-micron PVA particles. An ice pack was applied during embolization (Figures 4A, 4B). Manual compression was applied to the puncture site, requiring 30 minutes of pressure. The patient made an uneventful recovery and, after physiotherapy, regained knee movement from 5 to 110 degrees of flexion with no pain or swelling.

## Discussion

Delayed presentation of recurrent hemarthrosis after total knee replacement is rare. If left untreated, it can impair knee function, leading to painful stiffness and limited motion. Barrientos et al. [8] proposed an algorithm for managing recurrent hemarthrosis after knee replacement. Conservative management, including rest in a knee brace, ice application, and elevation, should be attempted first. A detailed history to identify any blood dyscrasias or anticoagulation use should be taken, and X-ray examination of the knee should be performed to exclude fractures and prosthetic loosening. Aspiration is mandatory for diagnosing hemarthrosis and for ruling out infection by sending the aspirated fluid for culture and sensitivity testing [6,9].

When conservative treatment fails, imaging modalities such as CT angiogram, MRI angiogram (MRIA), and Doppler ultrasound studies can be employed. Tomaszewski et al. [10] described the vascular anatomy of the knee, noting three levels of popliteal artery division. Type I occurs proximal to the knee joint, Type II at the knee joint, and Type III below the knee, with Type I being the most common, occurring in about 95% of cases. The danger zone for vascular injury is located between the 11 o’clock and 3 o’clock positions on the postero-lateral aspect of the tibial plateau [11]. CT angiograms are easy to perform and can clearly delineate active bleeding points. MRI angiograms provide detailed images of vascular anatomy and valuable information about soft tissue, especially synovial tissue pathology. However, the disadvantage of MRIA is that it can produce artifacts due to the metal knee prosthesis [12]. Doppler studies are particularly useful for ruling out arteriovenous fistulas [13].

Several studies in the literature have explored the use of selective genicular artery embolization to stop active bleeding from synovial hypertrophy in the treatment of recurrent hemarthrosis. Park et al. [14] reported that in their study of seven cases with recurrent hemarthrosis treated by embolization, six patients experienced complete resolution of symptoms. Luyckx et al. [15], in a retrospective study of 31 patients (39 embolization procedures) with recurrent hemarthrosis after knee arthroplasty, observed symptomatic improvement in 26 of 31 patients (84%). Power et al. [16] studied 13 consecutive patients (14 genicular artery embolization procedures) with recurrent hemarthrosis after knee replacement using 355 to 500 µm PVA particles, reporting a clinical success rate of 85.7%.

Arthroscopy or open arthrotomy is another option in the management of recurrent hemarthrosis after total knee replacement. These procedures are mainly indicated in patients with synovial hypertrophy [17], impingement, or pigmented villonodular synovitis [18], especially when angiographic studies are negative or when embolization has failed.

In our study, the second patient was on rivaroxaban, but hemarthrosis developed only six months after the knee replacement. The first patient had no risk factors for hemarthrosis. In both cases, knee aspiration was performed to confirm the diagnosis of hemarthrosis, and infection was ruled out by sending the aspirated fluid for culture and sensitivity. Both patients underwent CT angiogram followed by selective embolization, with excellent outcomes after a period of conservative management.

## Conclusions

Spontaneous hemarthrosis of the knee after total knee replacement is an unusual complication. Although uncommon, when it does occur, most patients respond well to conservative treatment approaches. However, if the bleeding recurs, it becomes crucial to evaluate for any underlying factors, both systemic and localized, that could be contributing to the issue.

A thorough diagnostic workup, including CT angiogram, MRI angiogram, or Doppler studies, is often necessary to pinpoint the exact source of the bleeding. If the bleeding source is identified, one effective treatment option is selective arterial embolization, particularly targeting the geniculate arteries. This procedure involves blocking the blood vessels responsible for the recurrent bleeding, thereby offering a safe and targeted approach to managing the condition.
